# Supplementary material for: Normative modelling of molecular-based functional circuits captures clinical heterogeneity transdiagnostically in psychiatric patients
Source: Commun Biol. 2024 Jun 5;7:689. doi: 10.1038/s42003-024-06391-3 (PMC11153627; doi:10.1038/s42003-024-06391-3)
Supplement: Supplementary file 2 — Supplementary material [file 42003_2024_6391_MOESM2_ESM.pdf]

Normative Modelling of Molecular-based Functional Circuits Captures Clinical Heterogeneity Transdiagnostically in Psychiatric Patients

Timothy Lawn[1], Alessio Giacomel[1], Daniel Martins[1,2], Mattia Veronese[1,3], Matthew Howard[1], Federico E. Turkheimer[1], Ottavia Dipasquale[1,4]

[1] *Department of Neuroimaging, Institute of Psychiatry, Psychology and Neuroscience, King's College London, London, UK*

[2]*Division of Adult Psychiatry, Department of Psychiatry, Geneva University Hospitals*

[3] *Department of Information Engineering, University of Padua, Italy*

[4]*Department of Research & Development Advanced Applications, Olea Medical, La Ciotat, France*

Supplementary Figures

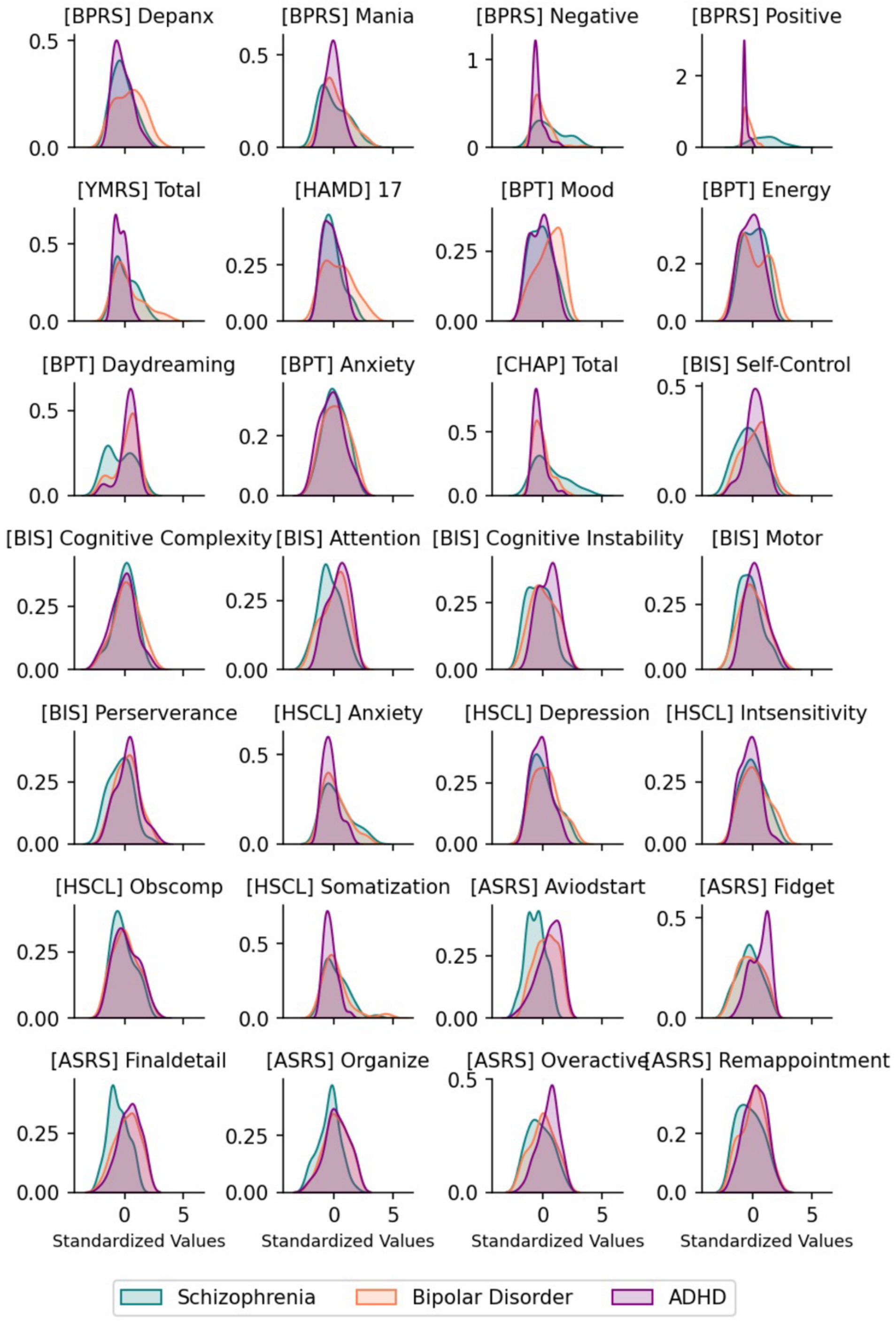

Supplementary figure 1: Normalised distributions of each psychometric measure for the different clinical cohorts.

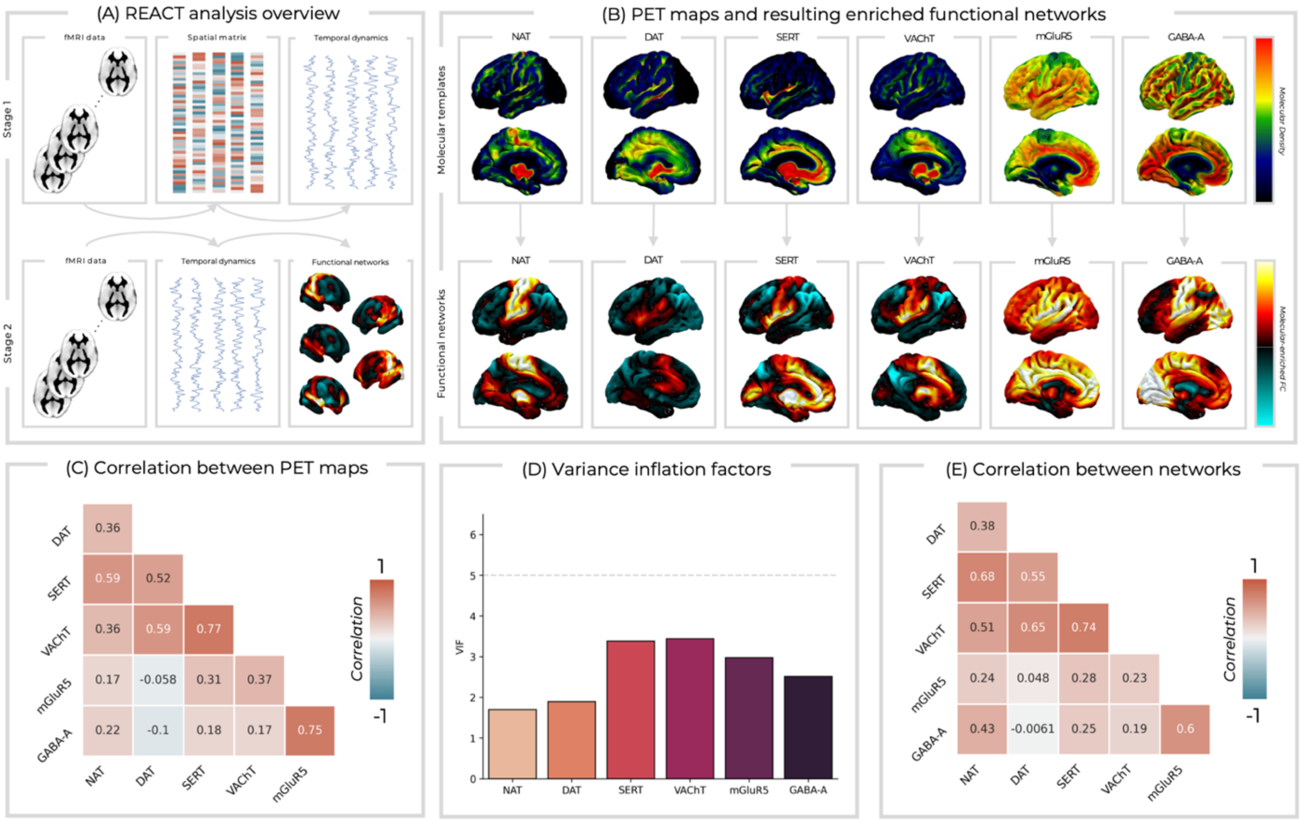

Supplementary Figure 2: (A) An overview of the REACT methodology. In the first general linear model (GLM) of the REACT analysis, the different molecular templates are spatially regressed against each subject's fMRI data, resulting in a time series capturing the spatial dynamics within each system. These time series are then used within the second GLM where they are regressed against the BOLD time series from each voxel in the fMRI data, resulting in molecular-enriched functional networks. (B) The PET maps utilised within the REACT analyses (top row) and the resultant molecular-enriched networks (bottom row). (C) Correlation coefficients between each pair of PET maps. (D) Variance inflation factors (VIF) for each PET map are below the rule of thumb value of 5, suggesting a non-problematic level of collinearity within the REACT model. (E) The correlation coefficients between each pair of molecular-enriched networks.

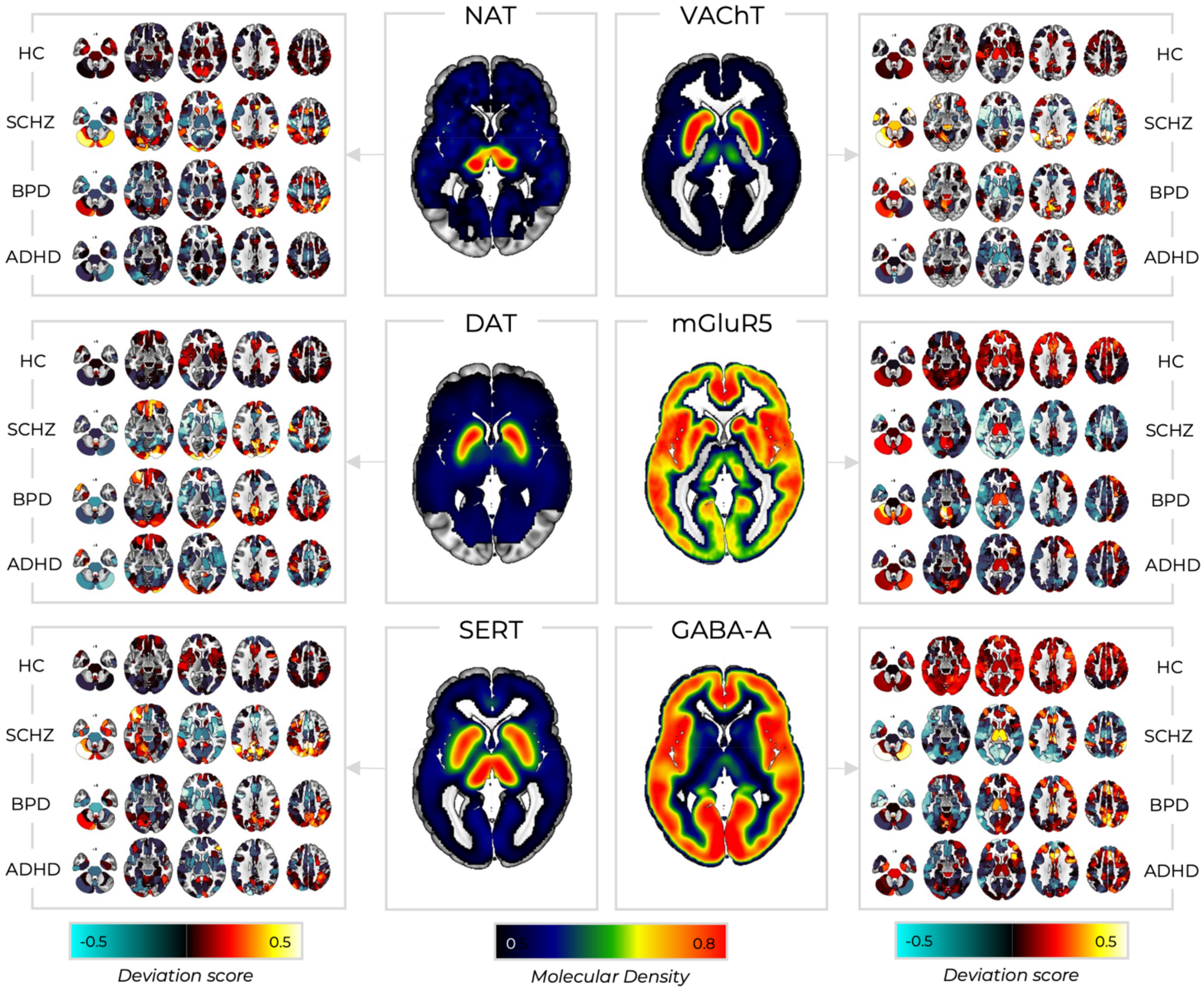

Supplementary Figure 3: (Middle) The 6 different molecular templates used to generate the molecular-enriched networks. (Right and left) the deviation maps for the test HC subjects (30%) and the three different diagnostic groups. Molecular density reflects normalised values which are standardized uptake value ratio (SUVr: DAT and vACHT) or binding potential (BP: NAT, SERT, GABA-A, and mGluR5) within the original PET data.

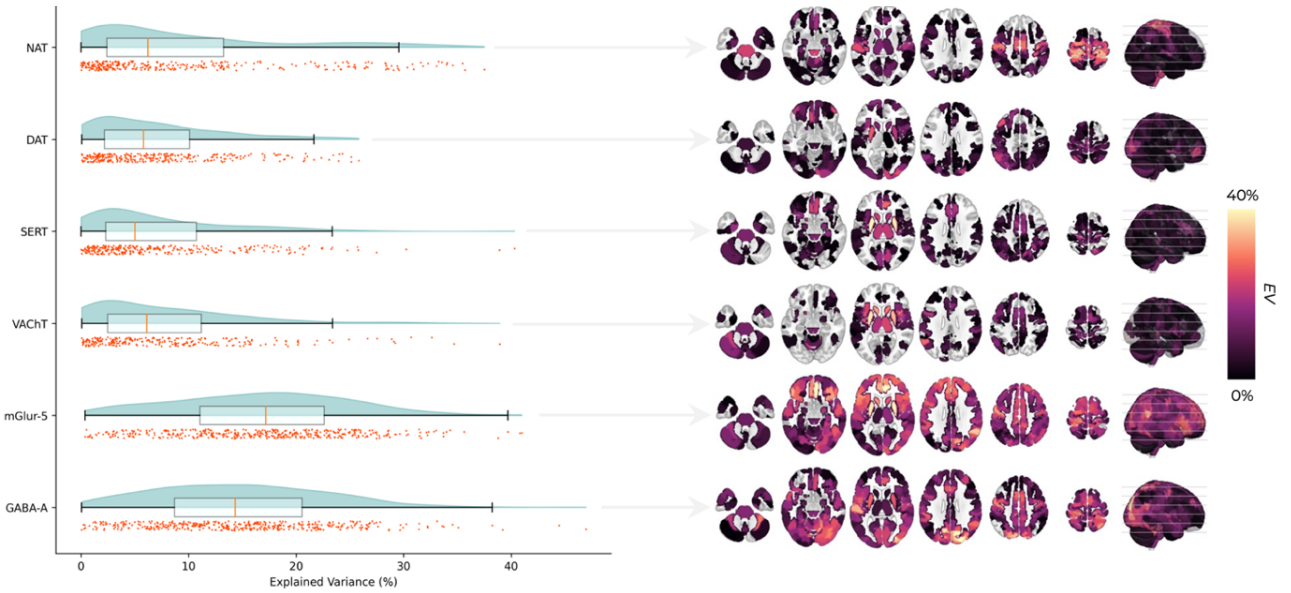

Supplementary figure 4: Models using age and sex as predictor variables explained a moderate amount of variance in the molecular-enriched networks for HC_{test} (left). Each raincloud represents the amount of variance explained across brain regions within each molecular-enriched system with each dot representing one brain ROI. The explained variance is also shown mapped back onto the brain for each set of molecular-enriched networks (right).

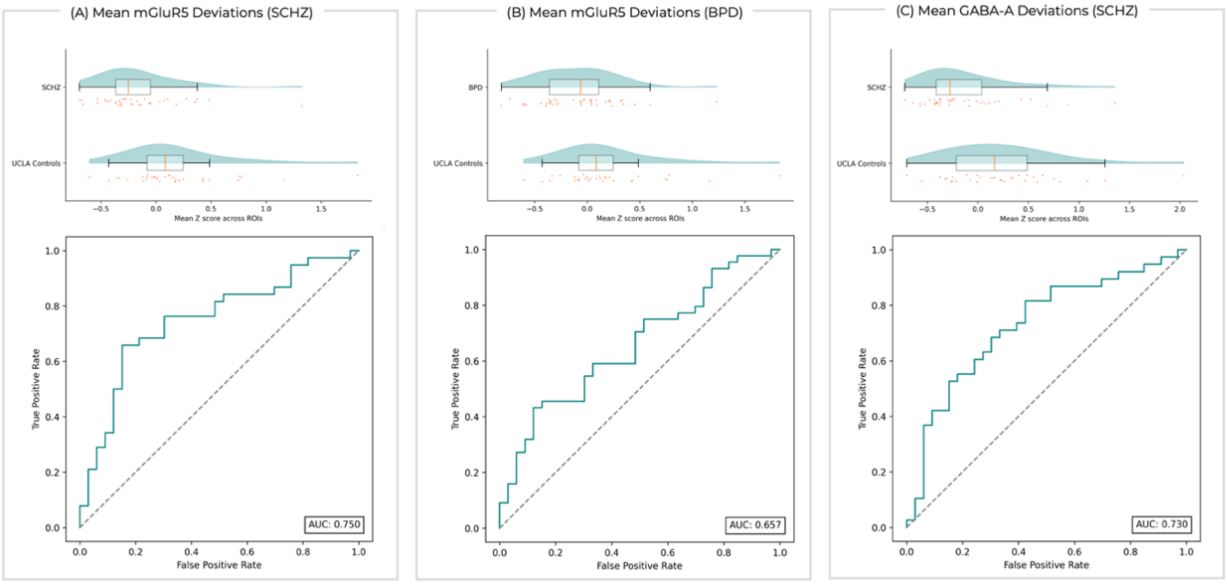

Supplementary figure 5: When comparing mean deviation scores for each subject across the different groups, significant differences were found for mGluR5 between healthy individuals from the UCLA dataset and patients with SCHZ (A) and BPD (B). Similarly, healthy individuals from the UCLA dataset differed from SCHZ patients for the GABA-A system (C). The top row shows raincloud plots of these mean deviation scores and the bottom row shows receiver operating curves demonstrating the predictive utility of these mean z values for classifying patients from controls.

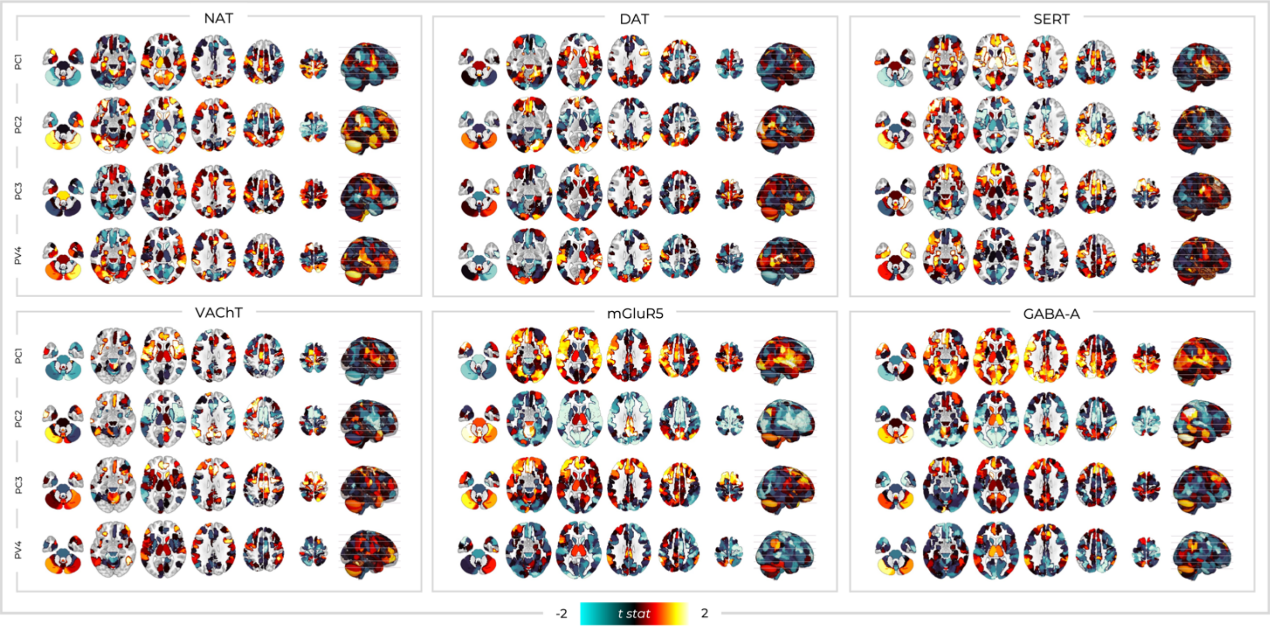

Supplementary figure 6: The t statistics from multiple-linear regression analyses examining the relationship between deviation scores and the four principal components at each ROI.

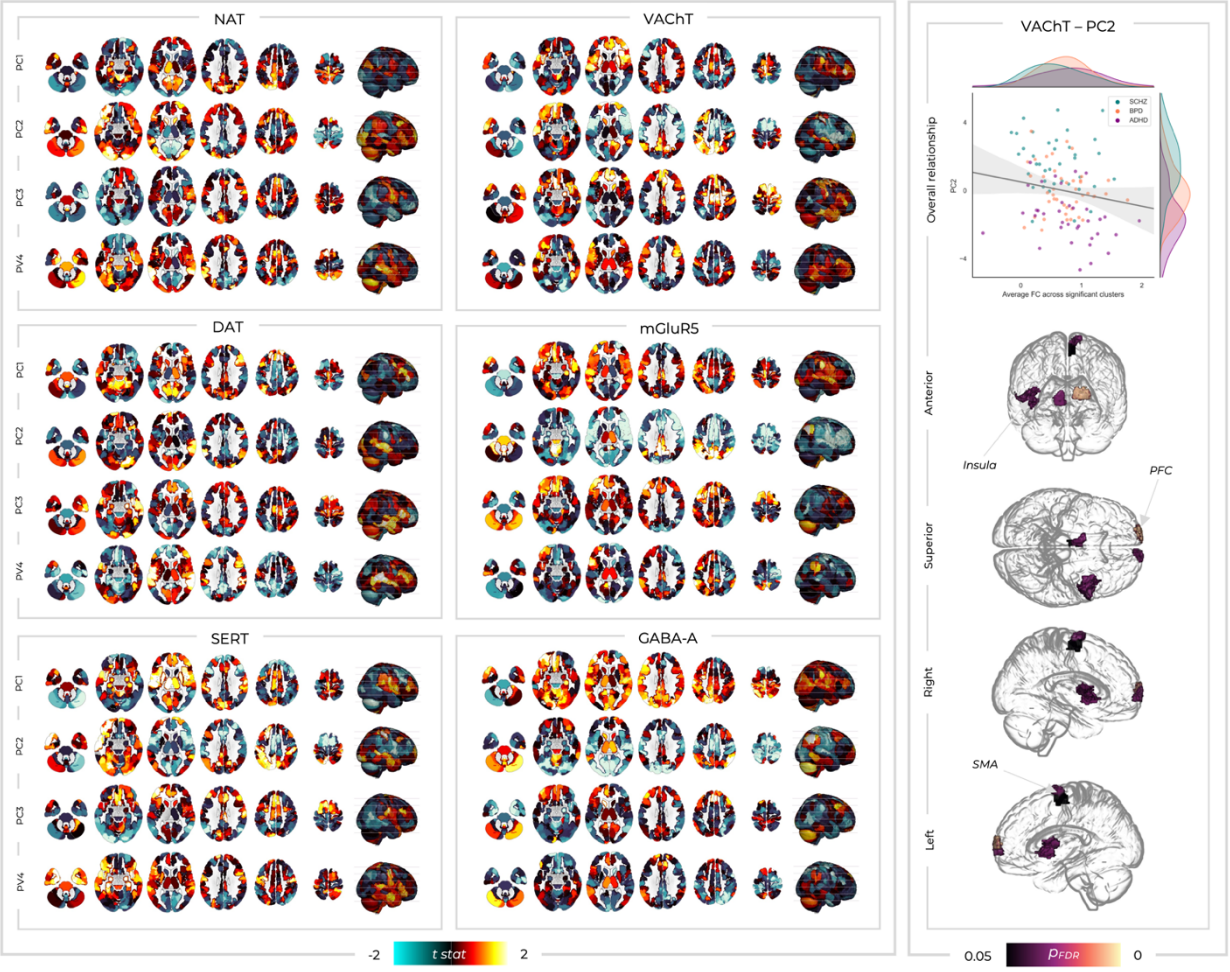

Supplementary figure 7: (Left box) The t statistics from multiple-linear regression analyses examining the relationship between molecular-enriched FC (as within a conventional REACT analysis) and the four principal components at each ROI. This allows for direct comparison with the normative modelling results running the same analysis but with deviations scores (figure X within main manuscript). (Right box) Only one of these deviation-symptom relationships was significant. Significant p_{FDR} values are shown for the relationships between PC2 and VAChT-enriched FC. The scatterplot shows this relationship with molecular-enriched FC averaged across all significant clusters. PFC; prefrontal cortex, SMA; supplementary motor area.

Supplementary Tables

Supplementary table 1: Summary statistics for within- and between-group similarity comparisons of psychometric data.

Comparison	Mean (SD)	N
SCHZ-SCHZ	0.129 (0.095)	38
SCHZ-BPD	-0.040 (0.038)	38
SCHZ-ADHD	-0.131 (0.098)	38
BPD-BPD	0.018 (0.032)	44
BPD-SCHZ	-0.040 (0.106)	44
BPD-ADHD	-0.0002 (0.118)	44
ADHD-ADHD	0.138 (0.120)	37
ADHD-SCHZ	-0.131 (0.079)	37
ADHD-BPD	-0.0002 (0.040)	37

Supplementary table 2: Within-group similarity (correlation of deviation scores between each pair of subjects across all 443 ROIs for each molecular system) for each receptor system and comparison across groups. Mean within-group similarity represents the mean correlation coefficient across every between-subject correlation within each group. Kolmogorov-Smirnov tests were conducted to compare these values from each clinical group to HC_{UCLA}. Asterisks denote significant differences following Bonferroni correction ($p < 0.05 / 18$).

Receptor	Group	Mean (SD) within-group similarity	KS statistic (vs HC)	*p* value (vs HC)
NAT	HC_{UCLA}	0.00 (0.15)	-	-
	SCHZ	0.11 (0.18)	0.27	0.00*
	BPD	0.06 (0.22)	0.23	0.00*
	ADHD	0.02 (0.18)	0.09	0.01
DAT	HC_{UCLA}	0.00 (0.15)	-	-
	SCHZ	0.10 (0.18)	0.24	0.00*
	BPD	0.05 (0.18)	0.13	0.00*
	ADHD	0.03 (0.17)	0.10	0.01
SERT	HC_{UCLA}	0.00 (0.18)	-	-
	SCHZ	0.13 (0.19)	0.26	0.00*
	BPD	0.07 (0.19)	0.14	0.00*
	ADHD	0.01 (0.18	0.05	0.54
VAChT	HC_{UCLA}	0.00 (0.16)	-	-
	SCHZ	0.15 (0.18)	0.35	0.00*
	BPD	0.06 (0.18)	0.15	0.00*
	ADHD	0.01 (0.17)	0.05	0.53
mGluR5	HC_{UCLA}	0.00 (0.15)	-	-

	SCHZ	0.13 (0.20)	0.35	0.00*
	BPD	0.05 (0.18)	0.14	0.00*
	ADHD	0.01(0.15)	0.03	0.90
GABA-A	HC_{UCLA}	0.01 (0.18)	-	-
	SCHZ	0.10 (0.21)	0.22	0.00*
	BPD	0.06 (0.20)	0.14	0.00*
	ADHD	0.03 (0.18)	0.05	0.38

Supplementary table 3: Correlations between transdiagnostic-group similarity and the four principal components for each different molecular system. Asterisks denote significant differences following Bonferroni correction ($p < 0.05 / 30$). Correlations were computed across all 443 ROIs and each analysis utilised all 119 patients.

Receptor	Component	Pearson's r	*p* value
NAT	PC1	-0.12	0.19
	PC2	0.24	0.01
	PC3	-0.04	0.70
	PC4	0.03	0.75
DAT	PC1	-0.13	0.16
	PC2	0.18	0.06
	PC3	0.04	0.67
	PC4	-0.01	0.89
SERT	PC1	-0.24	0.01
	PC2	0.34	0.00*
	PC3	-0.04	0.65
	PC4	0.07	0.46
VAChT	PC1	-0.19	0.04
	PC2	0.36	0.00*
	PC3	0.00	0.99
	PC4	0.07	0.44
mGluR5	PC1	-0.18	0.06
	PC2	0.34	0.00*
	PC3	0.07	0.47
	PC4	0.11	0.22
GABA-A	PC1	-0.18	0.05
	PC2	0.24	0.01
	PC3	0.03	0.74
	PC4	0.02	0.82

Supplementary table 4: Statistical tests comparing within- and between-group similarity of deviations within each different molecular system. A repeated measured ANOVA was first performed using the non-parametric test of Friedman, and post hoc analysis with Conover tests was conducted with a Bonferroni correction applied. Significant tests are highlighted with an asterisk.

Receptor	Comparison	*Repeated measures ANOVA*		Post-hoc comparisons	
		Chi-Square	*p*	*t*-stat	p_{bonf}
NAT	SCHZ-SCHZ vs SCHZ–BPD	38.90	< 0.001*	1.84	0.211
	SCHZ-SCHZ vs SCHZ-ADHD			6.08	< 0.001*
	SCHZ-BPD VS SCHZ-ADHD			4.24	< 0.001*
	BPD-BPD vs BPD-SCHZ	12.18	0.002*	1.07	0.868
	BPD-BPD vs BPD-ADHD			2.25	0.064
	BPD-SCHZ vs BPD-ADHD			3.41	0.003*
	ADHD-ADHD vs ADHD-SCHZ	1.95	0.378	0.70	1.00
	ADHD-ADHD vs ADHD-BPD			1.40	0.502
	ADHD-SCHZ vs ADHD-BPD			0.70	1.00
DAT	SCHZ-SCHZ vs SCHZ–BPD	22.47	<0.001*	2.868	0.016*
	SCHZ-SCHZ vs SCHZ-ADHD			4.703	< 0.001*
	SCHZ-BPD VS SCHZ-ADHD			1.835	0.221
	BPD-BPD vs BPD-SCHZ	7.81	0.020*	1.706	0.275
	BPD-BPD vs BPD-ADHD			1.066	0.868
	BPD-SCHZ vs BPD-ADHD			2.772	0.021*
	ADHD-ADHD vs ADHD-SCHZ	3.23	0.192	1.627	0.324
	ADHD-ADHD vs ADHD-BPD			0.116	1.00
	ADHD-SCHZ vs ADHD-BPD			1.511	0.405
SERT	SCHZ-SCHZ vs SCHZ–BPD	44.42	< 0.001*	2.98	0.012*
	SCHZ-SCHZ vs SCHZ-ADHD			6.65	< 0.001*
	SCHZ-BPD VS SCHZ-ADHD			3.67	0.001*
	BPD-BPD vs BPD-SCHZ	19.68	< 0.001*	0.21	1.00
	BPD-BPD vs BPD-ADHD			3.73	0.001*
	BPD-SCHZ vs BPD-ADHD			3.94	< 0.001*
	ADHD-ADHD vs ADHD-SCHZ	10.38	0.006*	2.79	0.007*
	ADHD-ADHD vs ADHD-BPD			2.79	0.007*
	ADHD-SCHZ vs ADHD-BPD			0.00	1.00
VAChT	SCHZ-SCHZ vs SCHZ–BPD	60.05	< 0.001*	2.98	0.012*
	SCHZ-SCHZ vs SCHZ-ADHD			7.69	< 0.001*
	SCHZ-BPD VS SCHZ-ADHD			4.70	< 0.001*
	BPD-BPD vs BPD-SCHZ	14.77	< 0.001*	1.07	0.868
	BPD-BPD vs BPD-ADHD			2.67	0.028*
	BPD-SCHZ vs BPD-ADHD			3.73	0.001*
	ADHD-ADHD vs ADHD-SCHZ	2.23	0.313	0.93	1.00

	ADHD-ADHD vs ADHD-BPD			1.51	0.405
	ADHD-SCHZ vs ADHD-BPD			0.58	1.00
mGluR5	SCHZ-SCHZ vs SCHZ–BPD	40.16	< 0.001*	3.67	0.001*
	SCHZ-SCHZ vs SCHZ-ADHD			6.31	< 0.001*
	SCHZ-BPD VS SCHZ-ADHD			2.64	0.030*
	BPD-BPD vs BPD-SCHZ	10.23	0.006*	1.60	0.34
	BPD-BPD vs BPD-ADHD			1.60	0.34
	BPD-SCHZ vs BPD-ADHD			3.20	0.006*
	ADHD-ADHD vs ADHD-SCHZ	9.243	0.010*	2.78	0.020*
	ADHD-ADHD vs ADHD-BPD			2.44	0.051
	ADHD-SCHZ vs ADHD-BPD			0.35	1.00
GABA-A	SCHZ-SCHZ vs SCHZ–BPD	26.11	< 0.001*	3.21	0.006*
	SCHZ-SCHZ vs SCHZ-ADHD			5.05	< 0.001*
	SCHZ-BPD VS SCHZ-ADHD			1.84	0.211
	BPD-BPD vs BPD-SCHZ	12.18	0.002*	1.07	0.868
	BPD-BPD vs BPD-ADHD			2.35	0.064
	BPD-SCHZ vs BPD-ADHD			3.41	0.003*
	ADHD-ADHD vs ADHD-SCHZ	2.81	0.245	1.63	0.324
	ADHD-ADHD vs ADHD-BPD			1.16	0.747
	ADHD-SCHZ vs ADHD-BPD			0.47	1.00

Supplementary methods

Participants and inclusion criteria

CamCAN

The data used here is from stage two of CamCAN, which contains a smaller subset of 700 individuals who met the eligibility criteria to proceed from stage one, including 100 individuals from each decile (18-87 years old). These criteria included having no signs of diminished cognitive health (mini-mental stage (MMSE) < 24, severe memory defects, or consent difficulties), no signs of communication difficulties (hearing problems, insufficient English language capabilities, or visual acuity difficulties), no self-reported medical problems (Parkinson's disease, motor neurone disease, multiple sclerosis, cancer in the last 6 months, strike, encephalitis, meningitis, epilepsy, head injury, recent or uncontrolled high blood pressure, pregnancy, BPD, SCHZ, or psychosis), no mobility problems (restrictions that would prevent participation or inability to walk 10 meters), no substance abuse (past or current treatment for drug abuse, current drug usage, or refusal to answer questions regarding drug abuse), and no MRI safety contraindications (such as implanted devices, claustrophobia, or inability to lie still for an hour).

UCLA

All UCLA participants were aged between 21-50, had completed at least 8 years of formal education, had no significant medical illness, were adequately cooperative, had visual acuity 20/60 or better, tested negative for drugs of abuse (Cocaine; Methamphetamine; Morphine; THC; and Benzodiazepines), were not pregnant, were not left-handed, did not have a history of head injury with loss of consciousness or cognitive sequelae, and did not have MRI contraindications (e.g. claustrophobia or metal in body). Participants in the healthy group [N = 130] were excluded if they had lifetime diagnoses of a psychiatric disorder. This included screening for sub-threshold ADHD using the Adult ADHD Interview and defined as 4 or more ADHD inattentive or hyperactive/impulsive symptoms in either childhood or adulthood. Each of the patient groups (SCHZ [N = 50], BPD [N = 49], and ADHD [N = 43]) excluded anyone with one of these other diagnoses. Stable medications were permitted for the patients.

Diagnoses for the clinical cohorts followed the Diagnostic and Statistical Manual of Mental Disorders (Fourth Edition)(1), utilising the Structured Clinical Interview for DSM-IV (SCID-I (2)) supplemented by the Adult ADHD Interview (a structured interview form derived from the Kiddie Schedule for Affective Disorders and Schizophrenia, Present and Lifetime Version (KSADS-PL)(3)). Interviewers were required to meet minimum standards of acceptable symptom agreement (overall kappa of .75, a kappa specificity of .75, kappa sensitivity of .75, and .85 kappa for diagnostic accuracy). Diagnostic and Symptom elicitation skill was also assessed with the SCID Checklist of Interviewer Behaviours (4) and the Symptom Checklist of Interviewer Behaviours (5). Ongoing quality assurance checks were conducted to ensure sufficient symptom agreement was being met.

Clinical and behavioural data

UCLA

A comprehensive list of the behavioural assessments can be found in table 3 of the original manuscript (6). Here, we utilised symptom measures from the Young Mania Rating Scale-C (YMRS), Hamilton Psychiatric Rating Scale for Depression (HAMD-17), Brief Psychiatric Rating Scale (BPRS), Hopkins Symptom Checklist (HSCL), and Adult Self-Report Scale v1.1 Screener (ASRS). Additional trait measures included in our analyses were the Barratt Impulsiveness Scale (BIS), Scale for Traits that Increase Risk for Bipolar II Disorder (BPT), and the Chapman Scale for Perceptual Aberrations (CHAP). The acronyms here correspond to those used in figures throughout the manuscript. Symptom and trait scores selected had data available across all three clinical groups, providing measures of symptoms that are conventionally associated more with one of the diagnostic groups, but with potential involvement within each. Participants with incomplete demographic or psychometric data were excluded. Where relevant sub-scores were available for these symptom and trait measures, we utilised these within subsequent analyses to preserve the rich dimensionality of this phenotypic data. Where sub-scores were not available, we used the total summary score. In total, this offered 28 measures.

We additionally examined within- and between-group similarity of these scores. First, we plotted density curves of each score split by clinical diagnosis to see how overlapping or non-overlapping they were. Next, we created a correlation matrix which examined how correlated each subject was to every other subject across all 28 clinical scores available. This essentially offers a metric of between-subject symptom similarity.

Imaging acquisition

CamCAN

All MRI datasets were collected at a single site (the Medical Research Council (UK) Cognition and Brain Sciences Unit) in Cambridge, UK) using a 3 T Siemens TIM Trio scanner (version syngo MR B17) with a 32-channel head coil. Functional MRI data were acquired with a T2* weighted GE EPI sequence with the following acquisition parameters: 261 time points, TR=1970ms, TE=30ms, flip angle =78°, FOV =192mm × 192mm, 32 axial slices, in-plane resolution = 3 × 3mm, slice thickness = 3.7mm, slice gap = 0.74mm. Additionally, a fieldmap was acquired: PE-GRE, TR=400ms, TE=5.19ms/7.65ms, 1 Magnitude and 1 Phase volume, 32 slices 3.7mm thick, 0.74mm gap, FA = 60°, FOV = 192 × 192mm, 3 × 3 × 4.44mm, TA=53s. A 3D MPRAGE was also collected: TR=2250ms, TE=2.99ms, TI = 900ms; FA = 9 deg; FOV = 256x240x192mm; 1mm isotropic; GRAPPA = 2; TA = 4mins 32s.

UCLA

MRI data were acquired on a 3T Siemens Trio scanner, located at the Ahmanson-Lovelace Brain Mapping Center (Siemens version syngo MR B15) and the Staglin Center for Cognitive Neuroscience (Siemens version syngo MR B17) at UCLA. Functional MRI data were collected using a T2*-weighted echoplanar imaging (EPI) sequence: 152 time points, TR = 2 s, TE = 30 ms, flip angle=90°, FOV = 192 x 192 mm, 34 slices, voxel size = 3 x 3 x 4mm, oblique slice orientation. An additional MPRAGE T1 was also collected: TR = 1.9 s, TE = 2.26 ms, FOV = 250 mm, matrix = 256×256, sagittal plane, slice thickness = 1mm, 176 slices.

Imaging pre-processing

The two datasets (CamCAN and UCLA) were used from their raw format and preprocessed for this work with nearly identical pipelines. FMRIPrep (version 20.2.5) was used to undertake initial processing steps with subsequent denoising, filtering, and registration implemented separately (7). The only difference was the utilisation of a field map to correct for field inhomogeneity in the CamCAN data which was not performed for the UCLA data.

The fMRIPrep pipeline first corrected the structural T1 images for intensity non-uniformity and extracted the brain (i.e., skull stripping). This extracted brain was then spatially normalised into standard space through non-linear registration to the MNI152NLin6Asym template using ANTs (8). fMRI data were corrected for motion using MCFLIRT ((9)) and co-registered to the T1 weighted image using boundary-based registration (BBR) with six degrees of freedom (10). For CamCAN participants only, a subject-specific field map was used to correct the distortion caused by field inhomogeneity (11, 12). fMRI data were also slice-time corrected, smoothed with an isotropic Gaussian kernel of 6mm FWHM (full-width half-maximum), and automatic removal of motion artefacts using independent component analysis (ICA-AROMA) was performed (13). Finally, using separate in-house scripts, subject-specific white matter (WM) and cerebrospinal fluid (CSF) masks were generated from the segmentation of structural images, eroded to reduce partial volume effects with grey matter (GM), co-registered to the subject-specific functional space, and used to extract and regress out mean WM and CSF signals from each participant's functional images. Finally, data were high-pass temporal filtered with a cut-off frequency of 0.001 Hz and normalised to the standard MNI152NLin6Asym template space at 2mm^3 resolution. Participants with head motion exceeding 0.5mm framewise displacement were excluded (14, 15). All fMRIprep quality control images and metrics were visually inspected for every subject, with participants additionally excluded for significant dropout in front-temporal regions or repeated registration failures.

Population-based molecular templates

We employed transporter and receptor density maps from the noradrenergic, dopaminergic, serotonergic, cholinergic, glutamatergic, and GABAergic systems. These are group average templates derived from healthy cohorts separate from the functional imaging datasets examined here. These have been widely utilised in our previous work (16–23), as well as by the broader imaging community (24, 25). Here, we chose to use transporters for the neuromodulatory systems as these provide a general measure of the innervation and influence of a given receptor system over a given region (26). The NAT template was derived from 10 healthy participants (M/F: 6/4, mean age (sd) = 33.3 (10.0) utilising S, S-[11C]O-methylreboxetine (27). The DAT template was derived using 123I-Ioflupane single-photon emission computerized tomography (SPECT) images from 30 healthy subjects (HS) with no evidence of nigrostriatal degeneration (28) (2 subjects < 35 years, 9 between 35-65 years and 15 > 65 years, no information available about gender). The SERT template was derived using [11C]DASB PET within 16 healthy individuals (internal PET database, M/F: 10/6, mean age (sd) = 59.4 (8.1)). The VAChT template utilised 18F-fluoroethoxybenzovesamicol PET within 6 healthy participants (3 male, 3 female, mean age (sd) = 67.0 (11.12) (29). [11C]flumazenil PET within 6 healthy individuals (male = 6, female = 0, mean age (sd) = 43 (4) (30) was used to produce the GABA-A template as described in (31). Finally, the mGluR5 template is from 31 healthy individuals

(male = 18, female = 13, mean age (sd) = 45.3 (18.2)) using [(11)C]ABP688 (32). Each template was first resampled to 2mm^3. Then, voxels within regions used as a reference for quantification of the molecular data in the kinetic model were replaced with the minimum value across all GM voxels in order to minimise the contribution of those regions without entirely excluding them from the models (i.e. occipital cortex for NAT and DAT as well as cerebellum for SERT, VAChT, and MGluR5). The values in these maps represent either standardized uptake value ratio (SUVr: DAT and vACHT) or binding potential (BP: NAT, SERT, GABA-A, and mGluR5). Finally, all templates were normalised by scaling image values between 0 and 1 to produce a normalised value of molecular density.

To examine collinearity between the receptor systems, we calculated the correlation coefficients between each pair of PET templates as well as their Variance Inflation Factors (VIF). VIF quantifies the severity of multicollinearity in a least squares regression analysis ($VIF = \frac{1}{1-R}$), with higher values (i.e., above 5) denoting strong collinearity.

Sensitivity analyses

In order to examine the benefit of utilising normative modelling over and above conventional REACT analyses, we conducted equivalent tests of between-group differences and deviation-symptom mapping on the molecular-enriched networks themselves rather than the deviation scores from entering these into the normative modelling framework. When running the same ANOVA on the molecular-enriched networks, no results were significant following multiple comparisons correction. We also repeated the deviation-symptom mapping analysis using the original molecular-enriched networks (SI- figure 7). Only the VAChT system showed positive results, with similar but more limited significant clusters. Specifically, these were in the right insular and left SMA as described above, but also bilateral PFC. No significant results were found for mGluR5 in this basic analysis.

References

1. J. Cooper, Diagnostic and statistical manual of mental disorders (4th edn, text revision)(DSM–IV–TR) Washington, DC: American Psychiatric Association 2000. 943 pp.\pounds 39.99 (hb). ISBN 0 89042 025 4. *The British Journal of Psychiatry* **179**, 85–85 (2001).
2. M. B. First, Structured clinical interview for DSM-IV-TR axis I disorders, research version, non-patient edition.(SCID-I/NP). *(No Title)* (2002).
3. J. Kaufman, B. Birmaher, D. A. Brent, N. D. Ryan, U. Rao, K-SADS-PL. *Journal of the American Academy of Child & Adolescent Psychiatry* **39**, 1208–1208 (2000).
4. J. Ventura, R. P. Liberman, M. F. Green, A. Shaner, J. Mintz, Training and quality assurance with the structured clinical interview for DSM-IV (SCID-I/P). *Psychiatry Research* **79**, 163–173 (1998).
5. J. Ventura, M. F. Green, A. Shaner, R. P. Liberman, Training and quality assurance with the Brief Psychiatric Rating Scale: "The drift busters." *International Journal of Methods in Psychiatric Research* **3**, 221–244 (1993).
6. R. A. Poldrack, *et al.*, A phenome-wide examination of neural and cognitive function. *Sci Data* **3**, 160110 (2016).
7. O. Esteban, *et al.*, fMRIPrep: a robust preprocessing pipeline for functional MRI. *Nat Methods* **16**, 111–116 (2019).

8. B. B. Avants, *et al.*, A reproducible evaluation of ANTs similarity metric performance in brain image registration. *NeuroImage* **54**, 2033–2044 (2011).
9. M. Jenkinson, P. Bannister, M. Brady, S. Smith, Improved Optimization for the Robust and Accurate Linear Registration and Motion Correction of Brain Images. *NeuroImage* **17**, 825–841 (2002).
10. D. N. Greve, B. Fischl, Accurate and Robust Brain Image Alignment using Boundary-based Registration. *Neuroimage* **48**, 63–72 (2009).
11. C. Hutton, *et al.*, Image Distortion Correction in fMRI: A Quantitative Evaluation. *NeuroImage* **16**, 217–240 (2002).
12. P. Jezzard, R. S. Balaban, Correction for geometric distortion in echo planar images from B0 field variations. *Magnetic Resonance in Medicine* **34**, 65–73 (1995).
13. R. H. R. Pruim, *et al.*, ICA-AROMA: A robust ICA-based strategy for removing motion artifacts from fMRI data. *Neuroimage* **112**, 267–277 (2015).
14. J. D. Power, K. A. Barnes, A. Z. Snyder, B. L. Schlaggar, S. E. Petersen, Steps toward optimizing motion artifact removal in functional connectivity MRI; a reply to Carp. *Neuroimage* **76**, 439–441 (2013).
15. J. D. Power, K. A. Barnes, A. Z. Snyder, B. L. Schlaggar, S. E. Petersen, Spurious but systematic correlations in functional connectivity MRI networks arise from subject motion. *Neuroimage* **59**, 2142–2154 (2012).
16. D. Boucherie, *et al.*, Modulation of functional networks related to the serotonin neurotransmitter system by citalopram: evidence from a multimodal neuroimaging study. 2022.10.20.512503 (2023).
17. M. Cercignani, *et al.*, Cognitive fatigue in multiple sclerosis is associated with alterations in the functional connectivity of monoamine circuits. *Brain Commun* **3**, fcab023 (2021).
18. O. Dipasquale, *et al.*, Unravelling the effects of methylphenidate on the dopaminergic and noradrenergic functional circuits. *Neuropsychopharmacol.* **45**, 1482–1489 (2020).
19. O. Dipasquale, *et al.*, Receptor-Enriched Analysis of functional connectivity by targets (REACT): A novel, multimodal analytical approach informed by PET to study the pharmacodynamic response of the brain under MDMA. *Neuroimage* **195**, 252–260 (2019).
20. T. Lawn, *et al.*, The effects of propofol anaesthesia on molecular-enriched networks during resting-state and naturalistic listening. *NeuroImage* **271**, 120018 (2023).
21. T. Lawn, *et al.*, Differential contributions of serotonergic and dopaminergic functional connectivity to the phenomenology of LSD. *Psychopharmacology (Berl)* **239**, 1797–1808 (2022).
22. D. Martins, *et al.*, A candidate neuroimaging biomarker for detection of neurotransmission-related functional alterations and prediction of pharmacological analgesic response in chronic pain. *Brain Commun* **4**, fcab302 (2022).
23. N. M. Wong, *et al.*, Differences in social brain function in autism spectrum disorder are linked to the serotonin transporter: A randomised placebo-controlled single-dose crossover trial. *J Psychopharmacol* **36**, 723–731 (2022).
24. J. Dukart, *et al.*, JuSpace: A tool for spatial correlation analyses of magnetic resonance imaging data with nuclear imaging derived neurotransmitter maps. *Human Brain Mapping* **42**, 555–566 (2021).
25. J. Y. Hansen, *et al.*, Mapping neurotransmitter systems to the structural and functional organization of the human neocortex. *Nat Neurosci* **25**, 1569–1581 (2022).
26. T. Lawn, *et al.*, From neurotransmitters to networks: Transcending organisational hierarchies with molecular-informed functional imaging. *Neuroscience & Biobehavioral Reviews* **150**, 105193 (2023).
27. S. Hesse, *et al.*, Central noradrenaline transporter availability in highly obese, non-depressed individuals. *Eur J Nucl Med Mol Imaging* **44**, 1056–1064 (2017).
28. F. J. García-Gómez, *et al.*, [Elaboration of the SPM template for the standardization of SPECT images with 123I-Ioflupane]. *Rev Esp Med Nucl Imagen Mol* **32**, 350–356 (2013).

29. M. Aghourian, *et al.*, Quantification of brain cholinergic denervation in Alzheimer's disease using PET imaging with [18F]-FEOBV. *Mol Psychiatry* **22**, 1531–1538 (2017).
30. J. F. Myers, *et al.*, Characterisation of the Contribution of the GABA-Benzodiazepine α1 Receptor Subtype to [11C]Ro15-4513 PET Images. *J Cereb Blood Flow Metab* **32**, 731–744 (2012).
31. J. Dukart, *et al.*, Cerebral blood flow predicts differential neurotransmitter activity. *Sci Rep* **8**, 4074 (2018).
32. J. M. DuBois, *et al.*, Characterization of age/sex and the regional distribution of mGluR5 availability in the healthy human brain measured by high-resolution [(11)C]ABP688 PET. *Eur J Nucl Med Mol Imaging* **43**, 152–162 (2016).
